# Discordant Evolution of the Adjacent Antiretroviral Genes *TRIM22* and *TRIM5* in Mammals

**DOI:** 10.1371/journal.ppat.0030197

**Published:** 2007-12-21

**Authors:** Sara L Sawyer, Michael Emerman, Harmit S Malik

**Affiliations:** 1 Division of Basic Sciences, Fred Hutchinson Cancer Research Center, Seattle, Washington, United States of America; 2 Division of Human Biology, Fred Hutchinson Cancer Research Center, Seattle, Washington, United States of America; Institute for Research in Biomedicine, Switzerland

## Abstract

TRIM5α provides a cytoplasmic block to retroviral infection, and orthologs encoded by some primates are active against HIV. Here, we present an evolutionary comparison of the *TRIM5* gene to its closest human paralogs: *TRIM22*, *TRIM34*, and *TRIM6*. We show that *TRIM5* and *TRIM22* have a dynamic history of gene expansion and loss during the evolution of mammals. The cow genome contains an expanded cluster of *TRIM5* genes and no *TRIM22* gene, while the dog genome encodes *TRIM22* but has lost *TRIM5*. In contrast, *TRIM6* and *TRIM34* have been strictly preserved as single gene orthologs in human, dog, and cow. A more focused analysis of primates reveals that, while *TRIM6* and *TRIM34* have evolved under purifying selection, *TRIM22* has evolved under positive selection as was previously observed for *TRIM5*. Based on *TRIM22* sequences obtained from 27 primate genomes, we find that the positive selection of *TRIM22* has occurred episodically for approximately 23 million years, perhaps reflecting the changing pathogenic landscape. However, we find that the evolutionary episodes of positive selection that have acted on *TRIM5* and *TRIM22* are mutually exclusive, with generally only one of these genes being positively selected in any given primate lineage. We interpret this to mean that the positive selection of one gene has constrained the adaptive flexibility of its neighbor, probably due to genetic linkage. Finally, we find a striking congruence in the positions of amino acid residues found to be under positive selection in both TRIM5α and TRIM22, which in both proteins fall predominantly in the β2-β3 surface loop of the B30.2 domain. Astonishingly, this same loop is under positive selection in the multiple cow *TRIM5* genes as well, indicating that this small structural loop may be a viral recognition motif spanning a hundred million years of mammalian evolution.

## Author Summary

The intrinsic immunity protein TRIM5α provides a post-entry defense against retroviral infection, which depends on its specific ability to recognize retroviral capsids. TRIM5α has been locked in genetic conflict with retroviruses throughout most of primate evolution, characterized by a higher than expected rate of amino acid change, referred to as positive selection. Here, we find that one of *TRIM5*'s closest human paralogs, *TRIM22*, has also undergone positive selection in primates. However, we find that its close linkage to *TRIM5* has resulted in an anti-correlated pattern of positive selection, with primate lineages generally showing positive selection in either *TRIM5* or *TRIM22*, but not both. Amino acid positions in TRIM22 found to be under positive selection are in remarkable proximity to the “antiviral specificity patch” previously described for TRIM5α. *TRIM5* and *TRIM22* evolution appears to be equally discordant in other mammals; the cow genome contains an expanded cluster of *TRIM5* genes and no *TRIM22* gene, while the dog genome encodes *TRIM22* but has lost *TRIM5*. Our analyses highlight *TRIM22* as bearing all the evolutionary hallmarks of a candidate intrinsic immunity gene.

## Introduction

Humans and other primates encode several intracellular proteins that can potently inhibit retroviruses after they have entered target cells [1–6]. One such protein, TRIM5α, exists in highly dynamic cytoplasmic structures [7] and intercepts retroviruses through recognition of the retroviral CA (capsid) protein assembled onto a viral core [8], leading to accelerated uncoating of the viral particle [9]. Human TRIM5α can block some retroviruses, but has insufficient activity against HIV [10,11]. However, the TRIM5α protein encoded by rhesus monkeys and some other primates efficiently blocks HIV infection [10,12–15]. Species specificity of TRIM5α for retroviruses can be altered by only a few amino acid changes in the coiled-coil and/or B30.2 protein domains [16–18]. Both of these domains have been subject to positive selection in primates [16], confirming that the ongoing host-virus “arms race” is leading to rapid change at viral interaction surfaces. Thus, the species specificity currently observed in this restriction system has presumably resulted from evolutionary pressure exerted by previous or ongoing infections [11,16,19,20].

The human genome contains approximately 70 genes of the *TRIM* family, which characteristically encode a tri-partite protein motif (TRIM) [21–23]. This motif consists of a “RING” zinc-coordinating domain, one or two zinc-coordinating “B-boxes,” and an alpha-helical “coiled-coil” motif (also referred to as the RBCC domains), whose order and spacing are conserved. RING domains are often associated with E3 ubiquitin ligases, and several TRIM proteins have been found to have such activity [23–25]. Some members of the TRIM family form homo- and hetero-multimers predominantly via their coiled-coil domains [21]. Most *TRIM* genes also encode a variable C-terminal domain, and in over half of them, including the TRIM5α protein isoform of *TRIM5*, this is a B30.2 domain. While RING, coiled-coil, and B30.2 domains are also found in other protein families, the B-Box is a unique and defining domain of the *TRIM* family. The function of the B-box is unknown, but it is essential for restriction by TRIM5α [26,27], and mutations in the B-box have significant effects on the half-life of the TRIM5α protein [28]. Although *TRIM* genes are scattered throughout the human genome, *TRIM5* sits in a small cluster of four closely related *TRIM* genes that also includes *TRIM6*, *TRIM34*, and *TRIM22*.

Most members of the human *TRIM* gene family remain functionally uncharacterized, or have so far tested negative for antiviral activity [29,30]. However, there are a few exceptions. The TRIM1 protein has been demonstrated to weakly restrict the murine retrovirus N-MLV [15,30]. There is mounting evidence that *PML (TRIM19)* encodes antiviral activity against diverse viruses, including herpes simplex type 1 (HSV-1), vesicular stomatitis virus (VSV), influenza A, and human cytomegalovirus (reviewed in [1,22]). Overexpression of TRIM34 has been shown to restrict HIV-2, SIVmac, and EIAV [29,30]. TRIM25 is involved in signal transduction leading to interferon production in response to RNA viruses [25]. Recent evidence has suggested that TRIM22 may also have antiviral properties, although there is some inconsistency between studies. For instance, overexpression of TRIM22 can inhibit spreading infection of HIV-1 in certain cell types, including macrophages [31], and TRIM22 may down-regulate transcription from the long terminal repeat promoter of HIV-1 [32]. However, TRIM22 does not restrict HIV-1 infection in alternate assays and cell types [29,30]. Like TRIM5α, TRIM22 expression is induced by interferon, as might be expected for an antiviral protein [31–33]. Of this small collection of possible antiviral *TRIM* genes, *TRIM34* and *TRIM22* sit directly in the *TRIM5* gene cluster.

The evolutionary “Red Queen” hypothesis addresses proteins which, like TRIM5α, are directly involved in antagonistic interactions with another genetic entity [34]. Under this hypothesis, TRIM5α will be continually selected for protein innovation in order to maintain fitness relative to retroviruses. A common measure for quantifying protein evolution is the dN/dS parameter, which summarizes the rate of amino acid–altering DNA changes relative to the baseline of “silent” DNA changes [35]. By looking at the DNA sequence of *TRIM5* from multiple species, we were able to conclude that this gene has experienced accelerated protein evolution because of high dN/dS ratios [16]. In individual populations (like humans) the actual mechanism of positive selection is the selective sweep, where an advantageous mutation rises in frequency in the population by the forces of natural selection. However, this mutation will not rise in frequency alone, but will commonly bring along with it proximal mutations (good, bad, or neutral) as “hitchhikers.” Since *TRIM5* has been under positive selection [16], we asked how this has affected its genomic neighborhood containing related *TRIM* genes with antiviral potential.

We show that the cow genome contains an expanded cluster of *TRIM5* genes and no *TRIM22* gene, while the dog genome encodes *TRIM22* but has lost *TRIM5*. In contrast, *TRIM6* and *TRIM34* have been strictly preserved as single gene orthologs in these genomes. Based on *TRIM22* sequence from 27 primate genomes, we find strong evidence of episodic positive selection in primate *TRIM22* as was previously observed for *TRIM5* [16]. However, we find that the evolutionary episodes of positive selection that have acted on *TRIM5* and *TRIM22* are mutually exclusive, with generally only one of these genes being positively selected in any given primate lineage. Finally, we find a striking congruence in the positions of amino acid residues found to be under positive selection in both TRIM5α and TRIM22, which in both proteins fall predominantly in the β2-β3 surface loop of the B30.2 domain.

## Results

### Dynamic Evolution of the *TRIM6/34/5/22* Gene Cluster in Mammals

**Figure 1:**
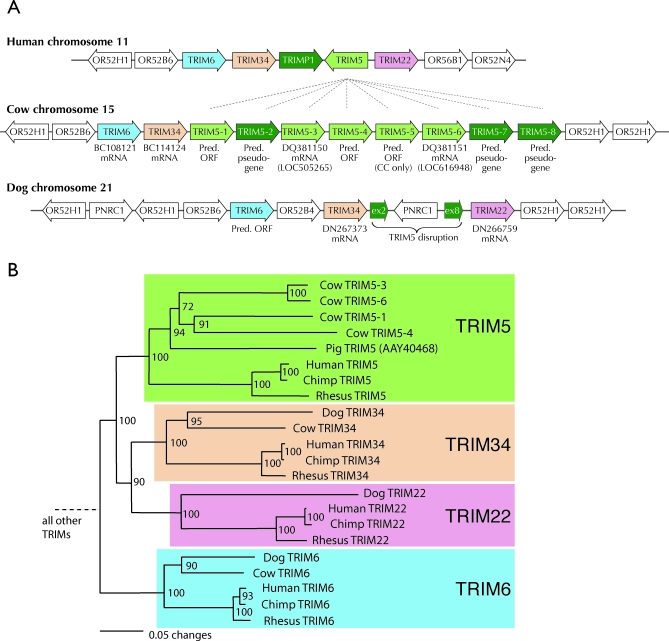
Evolutionary Dynamics of the TRIM6/34/5/22 Cluster (A) The cluster of *TRIM5*-related genes on human chromosome 11p is compared to the cow and dog clusters. In all three species the cluster is flanked by olfactory receptors (OR). Dark green genes are predicted *TRIM5* pseudogenes. The human *TRIM5* pseudogene (*TRIMP1*) is supported by mRNA AF230412 [21] and actually combines a region of *TRIM34* with a region within *TRIM5*. The remnants of two exons of the dog *TRIM5* gene are indicated as “ex2” and “ex8.” Expression evidence is listed below validated genes. See Appendix S1 for all dog and cow sequences. Genes not shown to scale. (B) A neighbor-joining tree based on RBCC protein domains shows the evolutionary relationship of predicted and validated genes. Cow *TRIM5–5* is not included because it encodes only a coiled-coil domain. The only known pig *TRIM5* gene is included. Colors correspond to those used in (A).

While the ∼70 *TRIM* genes are dispersed throughout the human genome, *TRIM5* sits in a cluster of four *TRIM* genes located at 11p15.4, which also includes *TRIM6*, *TRIM34*, *TRIM22*, and one pseudogene called *TRIMP1* (Figure 1A, top). Previous analyses have shown that these four *TRIM* genes are the closest human paralogs [29,36], indicating that this gene cluster probably arose through tandem gene duplication. We examined how *TRIM5* and its closest paralogs have evolved in mammals using available genome projects (Baylor Bovine Genome Project, [37,38]). The human, cow, and dog *TRIM6/34/5/22* gene clusters are illustrated in Figure 1A. In all three species, the gene cluster is flanked by tandem arrays of olfactory receptors, and is specifically preceded by *OR52H1* and *OR52B6* orthologs. We relied on neighbor-joining trees of RBCC protein sequences (Figure 1B) and DNA sequences (for pseudogenes and shortened genes, data not shown) to assign gene names by orthology, cognizant that the rapidly evolving B30.2 domain [16] may obfuscate true phylogenetic patterns. Bootstrap support for all four major clades is very strong (100%). Therefore we can clearly assign each cow and dog gene to a group orthologous to one of the four human *TRIM* genes.

The cow gene cluster on chromosome 15 contains eight *TRIM5* genes, five of which encode predicted or validated ORFs. An additional *TRIM5* ortholog, found on the cow chromosome 9, is predicted to encode an ORF of only the coiled-coil and B30.2 domains, although there is not yet evidence that it is expressed. This gene, *TRIM5–9*, is intron-less and is likely the result of a LINE-mediated cDNA integration (all cow and dog sequences can be found in Appendix S1). Inclusion on the tree of a *TRIM5* gene from the pig genome shows that the cow *TRIM5* expansion has occurred since cows diverged from their last common ancestor with pig (Figure 1B). Surprisingly, we do not find an ortholog of *TRIM22* in the cow genome (Figure 1A, middle line). Instead, the chromosome 15 gene cluster terminates into a long string of olfactory receptors (at least eight) before the closest contig gap in the genome assembly. BLAST searches of the cow genome and transcript databases also did not uncover a *TRIM22* ortholog, confirming our finding that the cow genome most likely lacks *TRIM22*.

In contrast, the dog cluster contains *TRIM22* but lacks *TRIM5*. The dog *TRIM5* gene has been disrupted by an insertion of the *PNRC1* gene. Copies of *PNRC1* reside both upstream and within the cluster, and these two *PNRC1* genes are 98% identical at the DNA level (479/489 bases identical), suggesting a recently shared gene ancestor. Cryptic, pseudogenized remnants of *TRIM5* exon 2 (encoding the RING and B-box2 domains) and exon 8 (encoding the B30.2 domain) were identified on either side of this gene (Figure 1A and Appendix S1). The absence of a functional *TRIM5* gene elsewhere in the dog genome was confirmed by BLAST analysis of genomic and transcript databases. Interestingly, most *in vitro* studies on TRIM-mediated retroviral restriction have relied on either feline or canine cells as a “blank slate” cell line that has little intrinsic restriction against retroviruses [39]. It is tempting to speculate that this phenotype is dictated by the loss of *TRIM5* genes in these species.

The opossum genome project is also suitably complete [40], but yielded no orthologs to these four genes. The most closely related *TRIM* gene in the opossum genome corresponds to human *TRIM39*, which in opossum is represented as an array of seven tandem, intron-less genes (data not shown). We can therefore date the *TRIM6/34/5/22* gene cluster to after the divergence of eutherian (placentals) and metatherian (marsupial) mammals around 180 million years ago [40], but before the divergence of the major eutherian groups containing dog, cow, and human beginning approximately 90–100 million years ago [41]. In support of the eutherian origin of this gene cluster, we also find no orthologs of these four genes in the chicken genome [42]. The most parsimonious explanation for these data is that a common *TRIM* ancestor gene initially gave rise to this cluster between 90 and 180 million years ago. Of these, *TRIM5* and *TRIM22* continued to be subject to gene gain and loss, while *TRIM6* and *TRIM34* remained more static.

### Positive Selection of Cow *TRIM5* Genes

**Figure 2:**
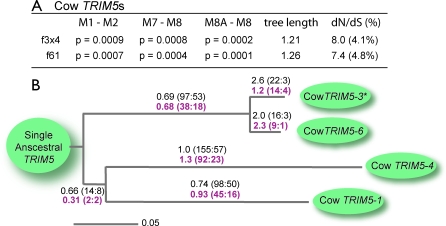
Natural Selection of Cow *TRIM5* Genes (A) The four full-length cow *TRIM5* genes or predicted ORFs (*TRIM5–1*, *TRIM 5–3*, *TRIM 5–4*, and *TRIM 5–6*) were analyzed for signatures of positive selection with PAML as described in the Methods section. Three different likelihood ratio tests between models allowing positive selection (M2 or M8) or only neutral/negative selection (M1, M7, or M8A) support the positive selection model (p-values all < 0.001), regardless of the model of codon frequency used (f3x4 or f61). With the f61 model, 4.8% of codons fall into a dN/dS category of 7.4. For more information on the codon evolution and frequency models, see the legend to Table 1. (B) A tree of the four cow *TRIM5* paralogs illustrates evolution since the divergence of these genes from their single common gene ancestor. dN/dS was calculated for each branch, and the actual numbers of replacement and synonymous DNA changes are given in parentheses (R:S). For instance, 22 replacement DNA changes and only three synonymous DNA changes have accumulated in the *TRIM5–3* sequence since the duplication that gave rise to it and to *TRIM5–6*. On each branch are listed values for the whole gene (top), and values calculated for the B30.2 domain alone (bottom, red). It appears that one more synonymous change occurred in the B30.2 domain of *TRIM5–3* than in the entire gene. While all synonymous changes in this lineage do occur in the B30.2, the estimate differs slightly depending on the dataset (3.2 in the full-gene analysis and 3.7 in the B30.2-only analysis) due to slightly different likelihood optima being reached. These values round to the integers “3” and “4.” The asterisk on *TRIM5–3* denotes that this gene was previously found to act as a retroviral restriction factor [36,43]. As with all highly related gene families, especially those arrayed in tandem, it is possible that gene conversion has obscured true phylogenetic relationships.

*TRIM5* duplications have been retained in the cow genome, and of these, *TRIM5–3* (previously known as LOC505265) has been shown to encode antiviral activity [36,43]. We can assess whether the *TRIM5* gene expansion in cows has been accompanied by evolutionary diversification of new paralogs by looking at the evolutionary signatures that these genes have accumulated since divergence from their common ancestor, the original cow *TRIM5* gene. We analyzed the evolution of the four full-length predicted or verified *TRIM5* genes (*TRIM5–1*, *TRIM5–3*, *TRIM5–4*, *TRIM5–6*). A multiple sequence alignment of these four genes was analyzed under various models of codon evolution to assess the support for positive selection*,* using a maximum likelihood approach as implemented in the PAML program [44]. Under this approach, there are some models of evolution (M1, M7, and M8A) in which codons are allowed to evolve under variable selective pressures but are constrained to neutral or negative selection (dN/dS ≤ 1). In alternate models (M2, M8), an adjustment is made so that a subset of codons is permitted to evolve under positive selection (dN/dS > 1). A likelihood ratio test is used to assess whether a model of positive selection fits the data significantly better than one of the “null” models. Regardless of models compared, or of the parameters defining codon frequencies in these models (f3x4 or f61), we find strong support for a sub-class of codons evolving under positive selection (p < 0.001, Figure 2A). We find that 4.8% of the *TRIM5* codons have an average dN/dS of 7.4. These data indicate that the four *TRIM5* paralogs have been under positive selection, potentially to diversify their capsid-binding function after expansion.

**Table 1:**
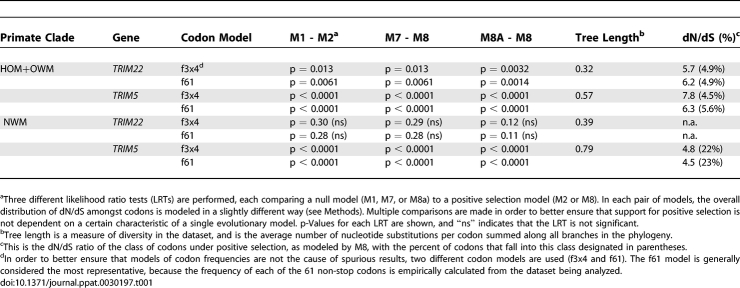
Positive Selection of TRIM22 and TRIM5

To determine whether the bovine *TRIM5–3* gene previously shown to restrict HIV [36,43] has been on a distinct evolutionary trajectory, we analyzed evolutionary signatures (dN/dS) along each branch of a tree representing these four cow paralogs (Figure 2B). Whole gene dN/dS values over 1.0 are considered extreme because they indicate that amino acid–altering mutations are being fixed in the gene even faster than neutral changes (in contrast, amino acid–altering DNA changes are usually poorly tolerated in protein-coding genes). Such large values of dN/dS represent the selection for protein innovation predicted to happen in arms race scenarios. The *TRIM5–3* branch has the highest value of dN/dS (2.6), and this gene has accumulated 22 non-synonymous DNA changes and only three synonymous changes since the duplication that resulted in *TRIM5–3* and *TRIM5–6.* The signature of *TRIM5–6* is nearly as high (2.0), and even higher than *TRIM5–3* when the B30.2 is analyzed separately (2.3 vs. 1.2). Signatures of positive selection along the *TRIM5–4* and *TRIM5–1* branches might be obscured due to the older divergence time of these genes (large dS values can make it difficult to detect positive selection). This analysis indicates that *TRIM5–3* may not be unique among cow *TRIM5*s in its antiviral potential, although only one of these may encode anti-HIV activity, as *TRIM5–6* has tested negative [36].

### Positive Selection of *TRIM22* in Primate Genomes

We have previously shown that *TRIM5* gene sequence has been shaped by positive selection for over 30 million years of primate evolution, even predating the evolutionary origins of primate lentiviruses [16]. We wished to address whether *TRIM6*, *TRIM34*, and *TRIM22* have also been evolving under a similar regime. Sliding window analysis of dN/dS along the length of these genes was used to identify gene regions subject to positive selection (data not shown, see [45] for method). This analysis was performed on *TRIM6*, *TRIM34*, and *TRIM22* gene sequence obtained from the three sequenced primate genomes: human, chimpanzee, and rhesus macaque [38,46,47]. We found no evidence for positive selection of either *TRIM6* or *TRIM34* (p > 0.05) but strong evidence for *TRIM22* (p < 0.05). Based on this result, we undertook a more extensive analysis of *TRIM22* in primate genomes. We sequenced the protein-coding sequence of *TRIM22* from six hominoids (HOM), seven old world monkeys (OWM), and eight new world monkeys (NWM) for a total of 21 full-length sequences representing 33 million years of primate divergence [48]. There is strong support for positive selection of *TRIM22* in the HOM+OWM clade (p < 0.02, Table 1). This signature of positive selection (5% of codons fall into a category of dN/dS = 6.2) is similar to the signature observed in a matched-primate analysis of *TRIM5* (6% of codons fall into a category of dN/dS = 6.3). This is remarkable, because *TRIM5* has one of the most extreme signatures of positive selection in the human genome [16].

Despite the strong signature of positive selection in the HOM+OWM clade, there was no support for positive selection of *TRIM22* in the NWM clade (p > 0.1, Table 1). The tree length (number of substitutions per codon) in the NWM clade (0.39) is greater than that of the HOM+OWM clade (0.32), suggesting that the lack of positive selection in the NWM clade is not a result of lower statistical power due to reduced evolutionary depth. The lack of positive selection in NWM *TRIM22* is in stark contrast to NWM *TRIM5*, for which there is strong support for positive selection in a matched-primate analysis (p < 0.0001). Therefore, we can conclude that the positive selection of *TRIM22* has predominantly occurred in OWM and hominoids, in contrast to the positive selection of *TRIM5,* which has occurred in all analyzed primates throughout their geographical ranges and evolutionary history.

### Disparate Evolutionary Histories of *TRIM5* and *TRIM22* in Primates

Another important means to elucidate the evolutionary history of a gene is by analyzing how dN/dS patterns have changed over distinct evolutionary lineages. For instance, our analysis of positive selection in *TRIM5* allowed us to conclude that TRIM5α's antiviral role is ancient but highly episodic [16]. We can now ask whether episodic selective pressures exerted by pathogens have simultaneously affected the evolution of both *TRIM5* and *TRIM22*; in this scenario, one might expect to find a correlation in dN/dS values between *TRIM5* and *TRIM22* over time. To test this hypothesis, we calculated dN/dS values along each branch of the primate phylogeny for both *TRIM22* and *TRIM5*, using the free-ratio model in PAML (see Methods).

**Figure 3:**
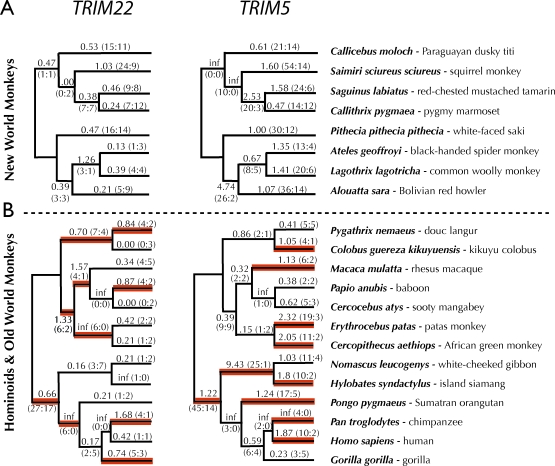
Disparate Evolution of *TRIM5* and *TRIM22* in Primates Branch values of dN/dS for the *TRIM22* and *TRIM5* genes are shown on the cladogram, along with numbers of replacement and synonymous changes (in parentheses, R:S) that occurred along each primate lineage. NWM (A) were analyzed separately from OWM+HOM (B). On the OWM+HOM cladogram, branches with the top ten values of dN/dS are highlighted in red. Branches with dN/dS of infinity (dS = 0) are included in the top ten if the ratio of R:S is greater than or equal to 4:0, since this ratio compares approximately to R:S ratios of the other top branches.

In the NWM clade, branch dN/dS values are almost uniformly lower for *TRIM22* than for *TRIM5* (Figure 3A). This is to be expected, since in NWM evidence for positive selection is strong for *TRIM5*, but not for *TRIM22* (Table 1). It is useful to ask whether fluctuations in branch dN/dS values truly represent changing selective pressures, or simply noise around an average value. We can address this issue by comparing the likelihood of a tree modeled with individual dN/dS values for each branch to the likelihood obtained when a single dN/dS is fitted onto the whole tree. Branch values on each of the *TRIM22* and *TRIM5* NWM trees are not significantly different from one another (Table 2), because a single universal dN/dS value cannot be statistically rejected (dN/dS_universal_ is calculated as 0.45 for *TRIM22* and 1.2 for *TRIM5*). Therefore, while two branches on the *TRIM22* NWM tree have dN/dS values greater than 1, we can conclude that these deviations from the average dN/dS are not statistically significant. Together, these patterns suggest that, in NWM, predominantly uniform selective pressures have acted on both genes, but that positive selection has played a major role in shaping only *TRIM5*. This implies a functional difference between these two genes in the primates of the Americas, and suggests that *TRIM5* may tend to be more commonly involved in pathogen protection than *TRIM22* in these primates, in either its canonical form or as TRIM-Cyp [49].

**Table 2:**
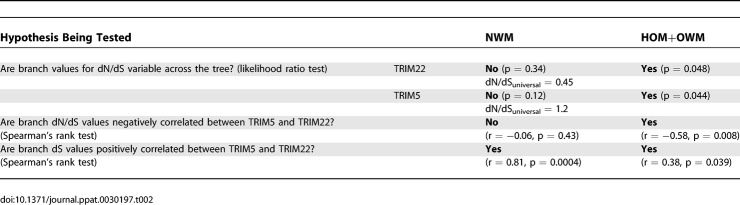
Statistical Analyses of Branch-Specific dN/dS Values

The situation is quite different in the HOM+OWM clade. In contrast to what we found in the NWM clade, episodic selection has acted on both *TRIM22* and *TRIM5* (p < 0.05, Table 2). Additionally, the branch-specific patterns of dN/dS for both *TRIM5* and *TRIM22* are quite different from one another (Figure 3B); branches with high values of dN/dS for one gene often have low values for the other gene. This is qualitatively illustrated by highlighting the ten branches on each tree that have the highest values of dN/dS (thick red branches, Figure 3B). Only two of these highlighted branches overlap between the two trees. The apparently inverse relationship between *TRIM5* and *TRIM22* dN/dS values can be tested with a rank-order correlation statistic, which supports a strong anti-correlation (r = −0.58, p < 0.01, Table 2). As a control, we find that dS values are correlated between the two genes (r = +0.38, p < 0.05, Table 2), as would be expected since dS is predominantly a function of evolutionary time represented by a given branch (the neutral mutation rate), whereas dN/dS represents the nature and intensity of selective constraint. dN/dS values for *TRIM5* and *TRIM22* are uncorrelated in NWM (r = −0.06, p = 0.43, Table 2), where branch-specific variability is not significant. In contrast to our initial hypothesis that episodic selective pressures may have simultaneously shaped these two closely related paralogs, this evidence suggests that *TRIM5* and *TRIM22* are anti-correlated in their evolutionary histories, and that usually only one of these genes appears to be subject to positive selection in any given lineage.

### Broad Expression of TRIM5α and TRIM22 in Humans

**Figure 4:**
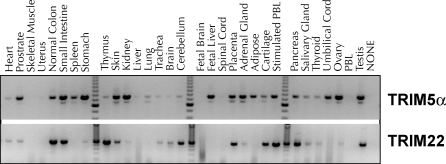
Expression Patterns of Human TRIM22 and TRIM5α Primers were designed to amplify ∼600 bp of the coiled-coil / B30.2 region of *TRIM5*α and *TRIM22* transcripts. These primers were used to amplify cDNA from 31 human tissues.

One easy explanation for the anti-correlation in their evolutionary patterns is that TRIM5α and TRIM22 target distinct classes of viruses. Indeed, in single cycle assays for retroviral infection, TRIM22 was not found to restrict any of the retroviruses that TRIM5α restricts [30], even when *TRIM22* orthologs from seven different primates were tested (our data not shown). To test the possibility that they might have distinct viral targets, we asked if these genes have evolved to produce unique expression patterns. Like many *TRIM* genes, *TRIM5* mRNA is alternatively spliced, and three different protein isoforms have been reported, each successively shorter from the C-terminus. Only a single isoform has been reported for *TRIM22*, which is similar in structure to the alpha isoform of *TRIM5* (TRIM5α), the longest *TRIM5* isoform and the only one with antiviral activity. Primers were designed to amplify *TRIM22* or *TRIM5*α transcripts from a panel of cDNA from different human tissues (Figure 4). We find that both *TRIM5α* and *TRIM22* are expressed broadly in humans, and that the tissues where *TRIM22* is expressed are for the most part a subset of tissues where *TRIM5*α is expressed. They are co-expressed in stimulated peripheral blood lymphocytes (PBL), which include the target cells for HIV and SIV, as well as in the testis, where heritable retroviral and retrotransposon insertions may provide a stringent selective pressure [11,16]. However, there also appear to be some tissues where only one of the two genes is strongly expressed. This opens the possibility that *TRIM5α* and *TRIM22* have evolved differential expression because they each target distinct pathogens which infect different tissues. However, the model that distinct pathogenic targets has led to the evolutionary anti-correlation also requires that these different viral classes never or rarely challenge the same host simultaneously, an assumption that is difficult to defend.

### Common TRIM22 and TRIM5α Amino Acid Sites Evolving under Positive Selection

The TRIM22 and TRIM5α proteins have a similar domain structure and share 58% amino acid identity (71% in the RING and B-box2 domains). These two proteins are most dissimilar in their coiled-coil and B30.2 domains, which include the retroviral recognition determinants mapped in TRIM5α [16–18]. We investigated whether these putative retroviral recognition determinants have also accumulated the signatures of positive selection in *TRIM22*, utilizing the HyPhy and PAML programs to identify codons evolving under positive selection (see Methods). Since positive selection is limited to the HOM+OWM clade, only the 13 OWM and hominoid sequences were analyzed, with one NWM sequence (titi monkey) included as an outgroup. Addition of a single NWM sequence improves the statistical power of the analysis (data not shown). For the large eighth exon, which encodes the B30.2 domain, six additional OWM and hominoid sequences were obtained and included in the analysis, in order to provide maximum depth and residue detection in this critical domain. There is strong evidence for positive selection in all of these sequence sets (p < 0.001 for all model comparisons).

**Figure 5:**
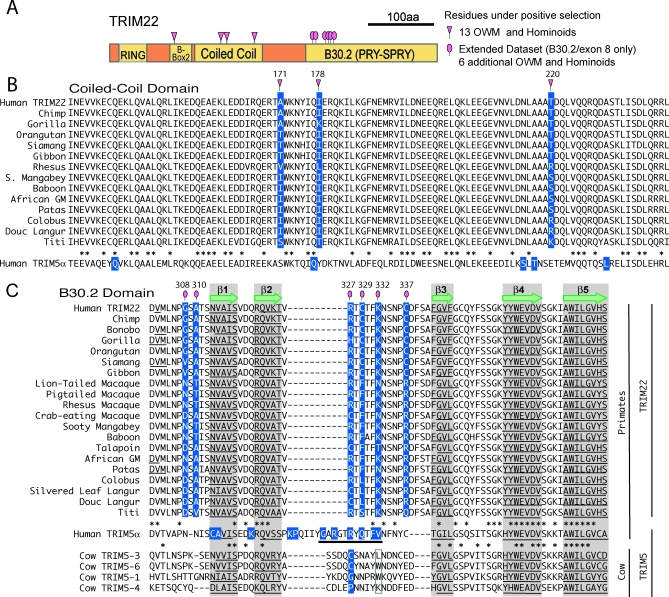
TRIM22 Amino Acid Sites Identified as Evolving under Positive Selection (A) A diagram of TRIM22 illustrates the amino acid positions highlighted as being subjected to positive selection (p > 0.95). Thirteen OWM and HOM sequences were analyzed for the first six coding exons (exons 2–7), with titi monkey included as an outgroup. For the large eighth exon, which encodes the B30.2 domain, six additional OWM and hominoid sequences were included in the analysis (listed in [C]). (B, C) Sequence is shown for the TRIM22 coiled-coil domain (B) and the first five beta-strands of the B30.2 domain (C). TRIM22 residues with strong support (p > 0.95) for positive selection are shown in blue highlight, with the symbols from (A) above the alignment. The human TRIM5α sequence is aligned, with residues previously found to be under positive selection [16] indicated in blue highlight. Residues indicated with asterisks are conserved with human TRIM5α. In (C), sequence motifs predicted to form beta strands are underlined (see Methods). Block arrows represent consensus beta strand positions, which are supported by crystallographic evidence for related B30.2 proteins [50,51]. The four cow *TRIM5* paralogs are aligned under the human sequence, with the single residue found to be under positive selection in a separate analysis of these genes highlighted in blue. The outlined box indicates an additional site identified when pig is included as an outgroup (several other sites are also identified in that analysis). Exact TRIM5-TRIM22 sequence alignment is somewhat uncertain in the region of the TRIM5α “patch” of positive selection (black horizontal bar and [16]) located in the β2-β3 loop.

Ten *TRIM22* codons have dN/dS values significantly greater than 1.0 (p > 0.95, Table S3), and these are schematically illustrated in Figure 5A. Of these, three codons lie in the encoded coiled-coil protein domain, in close proximity to the five positively selected sites previously documented in TRIM5α's coiled-coil domain (Figure 5B) [16]. The spatial similarity between sites identified for these two proteins suggests that there might be small segments of the coiled-coil that are especially relevant to viral interactions [17], even though it is predicted to form one long alpha-helical coil. The B30.2 domain consists strictly of tandem beta strands that fold into a beta-sandwich core [50,51]. Beta strands tend to be composed of conserved residues, while loops between beta strands are variable in both sequence and length [16,18,51]. Six of the ten positively selected sites in TRIM22 fall in the first part of the B30.2 domain, including four in the extended loop between beta strands 2 and 3 (Figure 5C). Surprisingly, the loop between strands 2 and 3 also corresponds to the location of the “patch” of HOM-OWM specific positive selection previously observed in TRIM5α (black horizontal bar [16]). Residues within this “patch” were shown to be the major specificity determinant of HIV recognition in TRIM5α [16,17,52]. Because of low sequence similarity in the loop between beta strands 2 and 3 (due in part to positive selection), exact TRIM5-TRIM22 sequence alignment is somewhat uncertain. However, it is intriguing that the TRIM22 residues identified show similar spacing to those of the TRIM5α patch. We can conclude that the positive selection of TRIM22 has been concentrated in the same regions as those responsible for retroviral specificity in TRIM5α.

We also analyzed the four full-length *TRIM5* genes from cow for codons under positive selection, where we find one codon identified with high confidence (p > 0.95). Amazingly, out of 470 codons analyzed, this site again falls directly in the β2-β3 loop of the B30.2 (Figure 5C). When the single known pig *TRIM5* gene is included as an outgroup, an additional codon in this region can be identified (outlined box, Figure 5C). This loop has therefore been targeted by positive selection in the *TRIM5* genes of both primates and cows. This, together with the identification of this loop in the analysis of primate *TRIM22*, illustrates the ancient importance of this small structural loop in the TRIM5/22-mediated arm of retroviral immunity.

## Discussion

### Ancient Signatures of Positive Selection in the Primate *TRIM22* Gene

We show here that powerful episodes of positive selection have acted on the *TRIM22* antiretroviral gene. The Red Queen hypothesis suggests that this signature could have arisen from millions of years of interaction between the TRIM22 protein and viral pathogens. While several other *TRIM* genes are known to encode at least weak antiviral activity, *TRIM5* is the only *TRIM* yet shown to possess such signatures [16,53], and now we find a second example with signatures of similar strength. It is possible that, while other *TRIMs* do have antiviral activity, *TRIM5* and *TRIM22* are unique in that they encode proteins that make direct physical contact with viral proteins, in contrast to indirectly affecting viral progression. While we know a significant amount regarding the importance and activity of TRIM5α against retroviruses, we know much less about the potential antiviral role of TRIM22. The present evolutionary analysis predicts that hominoid and old world monkey orthologs of *TRIM22* have antiviral potential (based on strong signatures of positive selection), that they operate through similar mechanisms as TRIM5α (based on congruence of positions of positively selected sites), but that their substrate specificity has been uniquely tailored over time (based on the anti-correlation between *TRIM5* and *TRIM22* selective signatures). Although no definitive targets for TRIM22 have yet been described, one would not necessarily expect TRIM22 to have activity against modern retroviruses since the agent that led to the selective events may not currently be circulating exogenously [11].

### Why Is *TRIM5* Copy Number So Dynamic over Evolutionary Time?

The cow genome encodes multiple *TRIM5* genes, while the dog genome encodes no *TRIM5* at all. It is easy to imagine why *TRIM5* may have duplicated so many times in cow, because multiple retroviral pathogens on different evolutionary trajectories essentially create multiple arms races in which *TRIM5* genes must simultaneously engage. Thus, increasing the number of *TRIM5* genes or alleles allows simultaneous selection for multiple retroviral affinities [20]. One possibility for the loss of *TRIM5* in dog is that another, redundant gene has largely taken over *TRIM5*'s antiretroviral function. In light of the current data it is tempting to speculate that this gene is *TRIM22*. Another possibility for the loss of *TRIM5* from the dog genome is that retroviral pathogens have not provided a constant selective force for maintaining this gene. We have previously argued that relaxation of selective pressures may result in the loss of functional *TRIM5* genes [19].

One of the *TRIM5* genes in cow, *TRIM5–3*, was shown to act as a retroviral restriction factor [36,43]. However, because the identity of this gene was at the time unclear, it was concluded by one group [36] that cows evolved a unique, non-*TRIM5* restriction factor from the *TRIM* gene family, in a scenario of convergent evolution in primates and cows. Here we definitively show that this gene is a cow ortholog of the human *TRIM5* gene, and that the acquisition of a novel *TRIM* restriction factor was not an independent event in cows [54].

### Anti-Correlated Evolution of Primate *TRIM5* and *TRIM22*

We find both similarities and differences in the evolutionary histories of *TRIM5* and *TRIM22* in primates. Despite the similar footprints of positive selection left on both genes, it appears that either *TRIM5* or *TRIM22* has be subject to strong (and therefore detectable) positive selection in any given primate lineage, but rarely both. One easy explanation is that TRIM5α and TRIM22 target distinct classes of viruses, or even different variants of the same virus. When one of these viral classes or variants is predominating in the environment, the corresponding *TRIM5* or *TRIM22* gene evolves under positive selection. However, this model requires the assumption that these different viral types never or rarely challenge the same host simultaneously. Instead, we favor the alternate possibility that this discordance in their positive selection is a direct result of tight genetic linkage due to their neighboring positions. As positive selection acts on one gene (e.g., *TRIM5*) and drives a particular allelic variant to higher frequency in a population, two consequences will arise: linked mutations in nearby genes will “hitchhike” along with this advantageous allele, and overall sequence diversity in neighboring regions will be reduced as this single allele dominates. This is commonly known as the Hill-Robertson effect [55]. The net result is that selection is weakened in surrounding regions, making it more difficult for a neighboring gene (in this case, *TRIM22*) to be simultaneously subject to positive selection. Indeed, several studies have pointed out that genetic linkage can limit the power of natural selection, subjecting genomic neighborhoods to more stochastic (rather than selective) changes [56–58].

Despite *TRIM5* and *TRIM22* being some of the most rapidly evolving primate genes, the possibility exists that a large portion of their adaptive landscape might still be unexplored if genetic linkage is dulling the power of selection on both. Additionally, since an adaptive event may require recurrent episodes of amino acid fixation, each gene may be slowed in its evolutionary “response time” to new pathogens. We propose that the Hill-Robertson effect may explain why large, related gene families (*TRIM*, olfactory receptors, etc.) tend to be broken up and scattered throughout genomes, because randomly occurring re-locations of single or groups of paralogous genes may be selectively favored in order to reduce recombinational “interference” between neighbors, and therefore to elicit maximal functional diversity from the family. A corollary of this prediction is that clusters that occur in genomic regions of high recombination may suffer fewer consequences of such interference. The *APOBEC3* cytidine deaminases may be an example of such a gene cluster, as even neighboring genes appear to have undergone simultaneous positive selection in certain primate lineages [45,59]. The *TRIM5* genes in cow may also be located in a recombinationally rich environment, since we observe simultaneous positive selection in several of these genes. Even with the limited information presented, there seems to be ample evidence of recombination in this region of the cow genome: loss of *TRIM22*, inversion of *TRIM5* orientation relative to human, and expansion of the *TRIM5* cluster since the cow-pig split.

### Patterns of Positive Selection Highlight Common Motifs for Substrate Recognition

Our findings suggest that the “rules” for TRIM restriction of viruses may be quite well defined. Selection for beneficial mutations at the host-pathogen interface is predicted to cause rapid amino acid change specifically at the protein-protein interaction interface between host and viral proteins. Positive selection has acutely targeted the coiled-coil and B30.2 domains of both TRIM5α and TRIM22. Specific residues in the B30.2 define HIV recognition [16–18], and the coiled-coil domain is also important for determining specificity to N-MLV [17]. The remarkable congruence in the positions of amino acid residues found to be under positive selection in both TRIM5α and TRIM22 suggests strongly that TRIM22 works through similar mechanisms of capsid recognition, and that the substrate recognition motifs will likely fall in the coiled-coil and B30.2 domains.

Surprisingly, our analyses continually identified the β2-β3 loop on the surface of the B30.2 domain [51,60] as an evolutionary hotspot, regardless of whether *TRIM5* paralogs from cow, or *TRIM5* and *TRIM22* orthologs from primates, were analyzed. This loop is also referred to as Variable Loop 1 (VL1), and together VL1 and VL6 make up the PRY binding pocket in the highly related TRIM21 for which the crystal structure has been solved [51]. VL1 is important for substrate binding in TRIM21 (TRIM21 binds circulating antibodies and can cause auto-immune disease) and bears a major disease mutation in TRIM20/Pyrin [51]. This suggests that VL1 is a malleable substrate specificity domain, and can be selected for and against certain molecular affinities. While other regions of the B30.2 also contribute to retroviral specificity [60], this study emphasizes the long-standing importance of the β2-β3 structural loop in substrate recognition.

## Methods

### Characterizing dog and cow *TRIM* gene clusters.

The UCSC and NCBI databases were queried with available *TRIM6/34/5/22* sequences to find all matches in the dog and cow genomes. These sequences were gathered and analyzed by phylogeny to establish gene families. PAUP (v4.0b10, [61]) was used to create and bootstrap neighbor-joining trees, and to create parsimony trees to verify relationships (data not shown). Pseudogenes and short ORFs (cow *TRIM5–2,-5,-7,-8,-9*) were confirmed as belonging to the *TRIM5* clade by DNA-based phylogeny (data not shown). To rule out genome assembly errors, the cow region spanning from *TRIM6* to *TRIM5–6* is supported by the BAC clone AC149772. The RefSeq gene track tool on the UCSC database (http://www.genome.ucsc.edu/) was used to identify neighboring genes. Naming of cow and dog predicted olfactory receptor genes (“OR”) is based on the closest match in the human genome.

### Sequencing of *TRIM22* coding sequences from cDNA and genomic DNA.

Primate *TRIM22* coding regions were sequenced either from genomic DNA (exons only) or from reverse transcribed mRNA. All primers and strategies used for amplification and sequencing are shown in Table S1. Primate DNA or cell samples were obtained from Coriell Cell Repositories (Camden, NJ) or from the Center for Reproduction of Endangered Species FrozenZoo Project (San Diego Zoo, San Diego, CA) and a list of primate species and sample numbers is shown in Table S2. PCR and RT-PCR products were sequenced directly, except in a few cases (denoted in primer table) where they were first cloned into the TOPO TA cloning vector (Invitrogen), followed by sequencing of independent clones. PCR from genomic DNA was performed with PCR Supermix High Fidelity (Invitrogen). RT-PCR from RNA was performed with the Superscript One-Step kit (Invitrogen) using RNA prepared with the RNeasy kit (Qiagen). Exon reads from genomic DNA were spliced together to create virtual transcripts. Exon structure was confirmed by full sequencing of RT-PCR products for the following primates: human, chimpanzee, gibbon, gorilla, orangutan, patas monkey, rhesus macaque, African green monkey, titi, tamarin, spider monkey, and woolly monkey. Alternately spliced transcripts of *TRIM22* were detected only in orangutan (data not shown). Virtual transcripts and cDNA sequences have been entered into the GenBank database (http://www.ncbi.nlm.nih.gov/Genbank/index.html), and accession numbers (EU124690– EU124716) are detailed in Table S2.

### PAML analysis of codon and lineage dN/dS values.

DNA sequences were aligned using Clustal_X [62] and PAL2NAL [63]. The codeml program in the PAML 3.14.1 package [44,64] was used to obtain maximum likelihood estimates for different models of codon evolution (see next section). The phylogeny of primate sequences was modeled as the currently accepted relationship for primates [48], which is the same tree as is derived from the *TRIM22* and *TRIM5* sequences with the exception of a few unresolved nodes. In order to ensure convergence of parameter optimization, each simulation was run with multiple seed dN/dS values. Each simulation was also run with two different models of codon frequencies, one referencing a 3x4 codon frequency table, and one in which the frequency of each of the 61 non-stop codons is empirically derived from the dataset. Identification of codon positions subject to positive selection was performed with codeml and with the random effects likelihood (REL) method of the HyPhy program [65,66]. Table S3 lists posterior probabilities for codon sites identified by PAML and HyPhy. A free ratio model (model = 1, one dN/dS per branch) was run in codeml to assess branch-specific values of dN/dS. This model also predicts the actual number of replacement and synonymous changes that occur along each branch. These values are in good agreement with changes assigned through parsimony (data not shown).

### Statistical analyses.

Likelihood ratio tests (LRTs) were performed to compare different models simulated with PAML. Codon models of neutral/negative selection (dN/dS of all codons bounded between 0 and 1) were compared to models of positive selection (models where an additional class of codons with dN/dS >1 is allowed). Three such comparisons were made: M1 vs. M2, M7 vs. M8, and M8A vs. M8, where M1, M7, and M8A are neutral/negative models and M2 and M8 are positive selection models. Models M1 and M2 assume that all codons fall into a few discrete categories of dN/dS, while models M7, M8, and M8A utilize a more fluid beta-distribution to model codon dN/dS values. Model M8A differs from M8 in that it allows an extra class of codons to evolve at dN/dS = 1. Model M8A was implemented as previously described [67]. LRTs were also used to assess whether a free ratio model (different dN/dS for each branch) fit the data better than a one ratio model (universal dN/dS for all branches). The Spearman's Rank Correlation test (performed with InStat, GraphPad Software, San Diego, CA) was used to determine the degree of correlation between branch values for the *TRIM22* and *TRIM5* datasets. For correlation of dS values, all branch data was used. For correlation of dN/dS values, a few branches where one or both genes had values of infinity (dS = 0) were not included, as values must be finite for this test. For both tests, one-tailed p-values were reported.

### Secondary structure predictions.

Protein sequences were submitted for secondary structure prediction on the JPRED server (http://www.compbio.dundee.ac.uk/~www-jpred/) [68].

### Expression profiles of *TRIM* genes.

Primers were designed to recognize *TRIM5*α and *TRIM22* transcripts and are listed in Table S1. In both cases, primers amplify about 600bp of the coiled-coil B30.2 gene region, and therefore the *TRIM5* primers specifically recognize the alpha transcript. These primers were used to amplify cDNA from 31 human tissues on PrimExpress Human Normal Tissue cDNA Panels (PrimGen, Bothell, WA). The cDNA on this panel has been optimized for equal amplification of a ubiquitously expressed microglobulin gene (PrimGen product literature). We utilized PCR Supermix (Invitrogen, 10790–020) for amplification reactions and amplified through 49 PCR cycles.

## Supporting Information

Appendix S1 Cow and Dog *TRIM6/34/5/22* Sequences (39 KB DOC)

Table S1 PCR, RT-PCR, and Sequencing Primers Used in This Study (66 KB XLS)

Table S2 Summary of Primate Samples Used in This Study (22 KB XLS)

Table S3 TRIM22 Codons under Positive Selection, Identified by HyPhy and PAML (16 KB XLS)
