# Supplementary material for: Discordant Evolution of the Adjacent Antiretroviral Genes TRIM22 and TRIM5 in Mammals
Source: PLoS Pathog. 2007 Dec 21;3(12):e197. doi: 10.1371/journal.ppat.0030197 (PMC2151084; doi:10.1371/journal.ppat.0030197)
Supplement: Supplementary file 1 [file ppat.0030197.sd001.doc]

APPENDIX 1: Dog and Cow sequences used in study

UCSB Assemblies Used: Dog = May 2005; Cow = August 2006

>DogTRIM6 GenScan gene prediction chr21_6.209 and XM_848205.1

ATGACTTCAGCGGTCCTGGTGGACATACGAGATGAAGTGACCTGCCCCATCTGCCTGGAGCTCCTGACAGAACCCGTGAGCATAGACTGTGGCCACAGCTTCTGCCAAGCTTGCATCATGGGAAATAGCACGGAGTTGGTGATGGGCCAAGACGGGGAAAGCAGCTGTCCTGTATGTCAGACCAGCTACCAGCCCGGGGACCTGCGGCCTAATCGGCACCTGGCCAACATAGCGGAGAGGCTCAGAGAGGTGGTACTAGGCCCTGGGATGCAGCTGAAAGTGACTCTCTGTGCACACCATGGGGAAAAGCTCCAGCTCTTCTGTAAGGAGGATGGGAAGCTTATATGCTGGCTTTGTGAGCGGTCTCAGGAGCACCGTGGTCACCACACGGTCCTTATGGAGGAGGTTGCCCACGAGTATCAGGAGAAGTTCCAGGAGTCTCTGGGGAAGCTGAGGCAAGAGCAACAGGAAGCTGAGAGACTAACAGCTGTTATCATAGGGAAGAGGACATCATGGAAGAATCAGATGGAACCTGAGAGACACAGGATCCAGACAGAATTTAATAAGTTGAGAAGCATCCTGGACAAAGAGGAGCAGCGACAATTGAAAAAGCTGGAGGAGGAAGAGAGGAAGGGCCTGAGTATTTTAGAAGAGGCTGAGAATGAGCTGGTCCAGCAGAGCGAAACACTGAGGGAGCTCATTTCTGATCTGGAGCTTCGGTGTCAGGGGTCAGCAATGGAGCTGCTGCAGGATGTGAGTGATGTCATGAAAAGAAGTGAGTTCTGGACCCTAAAGAAGCCAGAAGCTCTCCCTACCAAGCTGAAGAGTATGTTTCGAGTTCCAGATCTGAAAAAGATGCTGCGAGTGTTTAGAGAACTGACGGATGTCCAGAGCTACTGGGTGGACGTGACTCTGAATCCACACACGGCTAACTTAAATCTCGTCATGTCTAAAAACCGAAGACAGGTGAGATTTGTGGGTGCCAAGCTATCTGCGTCTTACCTGGAAGAGCATTATGACTGTGGTATCCTGGGCTCTCAGCACTTCTCCTCTGGGAAACATTACTGGGAGGTAGATGTGGCCAAGAAGACTGACTGGATCCTGGGGGTGTGCAGTAATTCAGTGGGACCAACGTACTGTTTCAGCCAGTTTACGAACAATCGAAATGTCTACTCCAGATATCAACCTCAGAGTGGATACTGGGTGATTGGGCTCCATCATAAACATGAATATAGAGCCTATGAGGAATCTTCTACTTCCCTGCTCCTCTCCATGATGGACTCCTGGCCTCTCCCGTATCCACGTCTTGGAGTTGGGGAAAGCCGGCCCTGTGCGTGGAAATCAGCGGGGAAAGCCGGCCCTGTGCGTGGAAATCAGCGCAGCACTGCAGGAAATCCCCGAGTTACAACAATTGGATGCCTGCGGTGA

>DogTRIM34 EST DN267373.1 (covers exon2 and part of 3) with rest picked up by BLAT using cow T34 protein

atggctttaaaaatcttgaacatacaggatgaggtatcctgtcccatctgcctggagctttcgacagaatcccggagtctagactgtggtcacagcttccgtcaaggctgcattgctgtgaataataaggaggcagagatcagccccagaggggaaagcacctgtcctctgtgtggtgtcagatactcactcagtaacctatggcataatcagcatctggtcaacatagtggagagaatcagaatgtttgagttgaacccagaagagttgctgaagagagacctctgtgtgcgccatggaaagaagctcctgctcttctgtaaggaggatagaaaggtcatttgttggctttgtgagcggtctcaggagcaccggggacaccaaacattcctcgtggaggaagtagtcaaggagtatcaggagaatctccaggcagctgtggagaggctgaggaaagagcagcagaaagctgagaagttggaaactgatatcagaggagagaggacttcCTGGAAGTACCAGATACAGACTGAGAGACAAAGAATTagaACAGAATTCAATCAGCTTAGAAGCATTTTGGACAGTGAAGAGcagAGAgttCTGCAAAAATTAGAAGAAGAGGAAAAGaggataCTGGATaacTTGGCTgagggtGAGGCTGAGTTGgctCAGCAGaaccagTTGgtgAAAGAGCTCATCTCAGATCTGGAGcatCGCAGTaaaTGGTCAACAGTGgacCTGCTGCAGAGTGAGATCTGGACACTGAAGAAGCCAAAAACTGTTTCCAAAagaCTGAAGagtataTTCcatgctCCAGATCTGagcacaATGCTGGTGGACATCACACTGAACCCAagcAACTTAAATCTAagcCTTGTCCTTTCAGAAGACCAGAGACAAGTGatgTCTGTGCCAATTTGGCCTGTTAAGtttagtAATTATGGTATCTTGGGCtccCAATATTTCTCCTCAGGGAAACACTACTGGGAAATAgagGTGTCCaagAAGactGCCTGGATCTTGGGGGTATACtctAGAaaaCGTtttcatAATATAaatcttggtGTTagaCAAggcACAgataatcaaAATgttTACTCTagaTACAGACCTCAGtgtGGCTACTGGGTCATTGGGTTAcagaatAAATTTgagTATaagGCCTTTGAGgagTCATCTACAtctcttcccatgGTCTTGACTattTCCATGgctGTTCCTCCCcatcgtattGGGGTTTTCCTAGACTATGAAGCAGGGattGTATCATTTcttAATGTCACAAACCACGGGTCACTCATCTACAAATTTTCTAAATGTtgcTTTTCTcacactgtgTATCCATACTTCAATCCTTGGaacTGCCCCgggCCTatcACCCTGTGCCCACCAcgcTCC

>DogTRIM22 XM_542402.2| PREDICTED: Canis familiaris similar to Tripartite motif protein 22 (RING finger protein 94; Staf-50; LOC485284). Supporting mRNA's are DN268615.1, DN265030.1, and DN266759.1.

ATGGATTTTCCAGCACAGGCAAACATACAGAAGGATGTGACCTGCCCCATTTGCCTCGAGCTTCTGACAATGCCTCTGAGCCTGGATTGTGGCCACAGTTTCTGCCAGGCTTGCATCACTGCAAAGAGCATGGAGTCAGGAACCCATCAAGGAGAGGAAGGCACCTGCCCAGTGTGCCAGTGCAAATACCAGTTTTGGAATCTCCGACCTAATCAGCCACTGGCAAACATAGCGAAGAAAGTCAGGGAGAACATGAGCCCACAGAAACAAAAGCTCTGTGAGGTCCATGGAGAAAAACTCCTGACCTTCTGTAAGGAGGATGGGAAGGCCATTTGCCAGCACTGTGCCCAGTCTGCAGAGCACCGTGGTCATCAAATATTCTTCATAGAGAAGGTCATCAAGGAATATCAGGAGAAACTCCAGGCAGCTCTGAAGAAGCTGAGGAAGGAGCATACAAAAGTGGAGGAGTTGGAAGCTGACATCAAAGAGAAGAGAGTTTCCTGGAAGAATCATATGCAGAGCGAGAGACAAAGGATTCTGAAAGGGTTTAATGAAATGAGAGACATTCTGAACAGTGAGGAGAAGAAGGTGCTGCAAAAGCTGGAGGAAGATGAAGTGAATGTGCTGGATAACTTGATAGTGGCCAGAGACCAGTTTGCTCAGCAGAAGCAATGCTTGAGAGAGCTCATCTCAGACATTGAGCATCAGATATGGGGGTCGTCAGTAGACACAGTGCAGGATATGATTAATGTCATGAAAAGGAGTGAGAGCTGGACCTTGAAAAAGCCAAACATTGTTTCCAAGAAACTGAAGAGTACATTCCTAATTCCGGATCTGAGTGGGATGTTGCAAGTGTTTAAAGAGCTGACAGAAGTCCAATGCTACTGGGTGGACGTGATGTTGAAGCCAGTCAATGCTATTTCGAATATTACCATTTCTGCAGATAAAAGACAAGTAACAGTTGTGCACAACTTCCCACTTAAAAGTATATATCCATTTGATTTCTCTGCTTTTGATATTTTGGGCTGCCAACATTTTTCCTCAGGGAAGTACTACTGGGAAGTAGATGTGTCTGGGAAGATTGCCTGGATCCTAGGGGTGTACAGTAAAGGAAGAAGCCTCAAGAGAAAAGGGGATTCTGGGTTTTTTTTTGATCCAAAAGTAAATCATCTAGATGTTTACTCCAGATTCAAACCTCAACTTGGTTACTGGGTTATTGGGCTACAGAATGAATCTGAGTATAAGGTCTTTGAGGAGTCTTCCACTTCTGATTCCAAGGTTTTGACTCTTTTCATGCCTGTTCCTCCCTGTCGTATTGGGGTTTTCCTAGACTATGAAGCACGCATTGTCTCATTTTTCAATGTCACAAACCATGGGTCACTCATCTACAAGTTTTCTGAGTGTCAGTTTTCTCAGACTACTTATCCATATTTCAATCCTTGGAACTGTCAATACCCTATCACTCTGTGCCCACCGAGTTCCTGA

>DogTRIM5 cryptic trim5 exon2 (pseudogenized)

TATGGCTTTTAAATTCCTAACATAGAGGGGGTGGTGATCTGCTCCATCTGCCTGGTCCTCCTGAGATAACCACCGAGCCTGGAATGTGGCCACAGCTTCTGTAAAGCCTGCTTTACTGCAGACAACAAGGAGTCCATGCCAAGCCAAGGAGGGAAGAACAGAAATTTCCCTGTATGCTGCATCAGTTACTGTGCTGCGGACCTGCGGCCTAACTGGCATGTGGTCGACATAGTAGAGTGGCTCAGAGAGCCCAAGGTGAGCCCTGAGGAGGGCCAGAAGAGAAATCTCTGTGAGCACCACAAAGAGAAACTCTTGTTCTTCTGTAAGGAGGATGGGAAAGTCATTTGCTGGCTTTGTGAGTGGTCTCAGGAGCACC

>DogTRIM5 cryptic trim5 exon 8 (pseudogenized)

ATTTCTACAGATCAAAGACAAATGAGATATGTGTTTCATTGGAAACTAGATAATGCATATAATAGCAGTTATGGTGTCCTTGGCTCCCTGGTCATTGGATTGGGGAAACATTACTGGGAGGTAGATcctggaggtataagatggaaaatgcccagaatacattatgaaatttcatgttagacatgattaaAATTATCAACATAGTTACTCTAGGTTTCAACCAAATATGACCACTGCATTAAACGGTGATGAATCAACTTTAtaatgcttttgaggagtcatccttctctgatctcctcatcttaatcctgaccctgcctgtcattctccatagGTTTTCCTAGACTATGAGGCTGGCATTGTCTTATTTTTCAATGTCACAAACTATGGGTTTCTCATCTATACGTTCTTTTCATTTTCCTCTTCTCAGGAAGCTTTTCCATATTTCAGCCCCATGAAATGGAATGACCCC

>CowTRIM5-1 Gnomon hmm95731 Predicted ORF. Also XM_864679.2| PREDICTED: Bos taurus similar to tripartite motif protein TRIM5, transcript variant 2 (LOC516599)

ATGGCTTCGGAAAAATTGGTGAGCCTACAGGATGAGGTGACCTGCCCAATTTGCCTGGAACTTCTAACAGAACCCCTGAGCCTTGACTGTGGCCACAGTTTCTGCCAAATCTGCATCACTGCAAACAGCAATGAGTCCCTGACTGGCCAAGAAAGGGCGAGAAAGTGCCCTGTGTGCAGAATCAATTATAAATCTGGAAAACTACGGCCTAATTGGCACCTGGCCAACATAGTGCAGAGGGTCAGGGAGGTCAAACTGAGCCTGGAGGAGCAAGAGAAACATCTCTGTGCTCACCATGGAGAGAAACTCCTGCTCTTCTGTGAGGAGGATGGGAAGGTCATTTGCTGGCTTTGTGAGCGGTCCCAGGAGCACCACGGTCACCCTACATTTCTCATGGAAGAGGTCGCCCAGGAGTACCAGAAGAAACTCCAGGCAGCTCTGGAGAGGCTGAAGCAGGAGCAGCAGGAAGCTGAGGAGTTGGAGTCTGACTTCAGAGAAGAGATTGCTGCCTGGAAGAATCAAACACAACATGAGAGACAAAATGTCCAGGCAGAGTTTAAAAAACTGAGAGAAATCCTGGACAGTGAGGAGAGGAAGGAACTACAAAAGCTGAAGGCTGAGGAGACAGCTATTCTGTGTACCATGGCAAACTCTGAGAATGAGCTGGTCCAGCAGAGCCAGTTGGTGAGAAATCTCATCTCAGATATAGAGCATCGTTTGCAGGGGTCAACAATGCAGATGCTGCAGGATGTGAATGACATCTTGAAAAGGAGTAAGACTTTGACTCTGAAGAAGCCAAAACCTGTTCCCAAAGAACAGAGAAGTGTGTTTCGAGCTCCTGATCTGAGAGATATACTGAGAGTGTTTAATGGGCTGAGATATGTGCAACGCTACTGGGTTCACGTGACCCTGAGTCATACAACAGGTAATCGAAATATCGCCATTTCTGCTGATCGAAGACAAGTGACATATGTGTACAAAGACCAAGGAAATAATCCACGCTGGAAACAAGTTTATGAAGATTATGGTGTCCTGGGATCCCCAGTTATCAAATCAGGGAGACATTACTGGGAGGTAGACGTGTCAAAAAAACGTGCCTGGATCCTGGGGGTATATGGTGAAAAACACGTTGAATATAATACAAAATGCCAACCTAAAAATGGCTACTGGGTCATAGGGTTAAGTTATGGTTCTGTATATAATGCTTTTGATGAGTCTTCCTCCTCAGATCCCATGATCTTAACCCTCTCCCTAAGTGTTCCTCCTCATCGTATTGGAATTTTCCTAGACTATAGTGCTCGCACTCTCTTATTTCTCAATGTCACAAACCACGGGTTTCTCATCTATAAATTCTCTTCATGTTCCTTTTCTCAAAAAATATTTCCGTATTTCAATCCTATGACCTGTAATGTCCCCATGAATCTGTGCTCGCCAAGCTCTTGA

>CowTRIM5-2 Gnomon hmm95349 predicted pseudogene

ATGGCTTCAGGAAAATCAGTGAACTTACAGGAGAAGTTCACCTGCCCCATTTGCCTGGAGCTTCTGACAGAACCCTTGAGCCTTGACTGTGGCCATAGCTTCTGTCAAGCCTGCAACACTGCAAACAACAAGGAGTCCGTGATTGGCCTAGAAGGGGAGAGAAGGTGCCCTGTATTCAGAATCAAGTATGAACCTNNGCGGCCTCATTGGCACCTGGCCAGCATAGTGGAGTGGCTCAGGGAGGTTAAGGTGAACCTGGAGGAGCAAGAGNNAGAAATCTGTCTGTGCCATGGAGAGAAAGTCCTGTTCTTCTGTGAGGAGGATGGCAAGGTCATTTGCTGGCTTTGCAAGTGATCACAGGAACACCATGGTCACCACACATTCCTCATGGAAGAAGTTGCCCAGAAGTACCAGTGGAAACTCCAGGAATTTTTGAAGAGGCTGAGGAGGGAGCAGCAGGGAGCTGAGAAACTGAAAGCTCAAATCAGAGGAGAGATTAATACCTGGAAGAATGAAATACAACACGAGAGAGAAAATATCCAGGCAGATTTTTAAAACCTGAGAGAGATCCTGAACAGTGAGGAGTTGAAGGAACGGCAAAATCTGAAGACTGAGGAGGAGGTTGGTCTATGTAACCTGGCTGACTCTGAGAATGAGCTGGTCCAGCAGAGCCAGTTGGTGAGAGATCTCATCTCAGATGTGGAGCATCATTTGCAGGAACCAAAAAACGAGATGCTGCAGCGGGATGTGCCACCCAGAAGCAGCTCAGCTGAAAGATTGGGGCAAGTAGAGCATGTGGGAAAAACTTCCAGAAGTACCATCTGGAAGCATCTAATTTCTAGTAACAGTGACATCTAA

>CowTRIM5-3 Gnomon hmm40532 predicted gene. Supported by DQ381150 mRNA sequence, which is slightly different. (Also referred to as LOC505265)

ATGGCTTCAGGAATCCTGATGAACATACAGGAGGAGGTGACCTGCCCCATTTGCCTGGAGCTTCTGACAGAACCCCTGAGCCTTGACTGTGGCCACAGCTTCTGTCAAGCCTGTATCACTGCAAACAACAAGGAATCCATAATTGGCCAAGAGGGGAAGAGAAGCTGCCCTGTGTGCAGAGTTAGTTTTGAGCCTGGGAACCTGCGGCCTAACAGGCATCTGGCCAATGTAGTGCAGAGGCTCCGAGAGGTTAAGGTGAGCCCAGAGGTGGACCAAGAGAGAAATCTCTGTGCTCACCATGGAGAGAAACTCCAGCTCTTCTGTAAGCAGGATGGGAAGGTCATTTGTTGGCTTTGTGAGCGATCTCAGGAGCACCGAGGTCACAACACATTCCTCATGGAGGAGATTGTGCCAGAGTACCAGAAAAAGCTCCAATCATGTCTGCAGAGGCTGAAGGGGAAAAAACAGAAAGCTGAGGAGTTGGAAATTAAAGTCAGAGAAGAGATGTCTACTTGGAAGATTCAAATAGAAAATGAGATACAAAATGTCCAGGGAAAGTTTACACAACTAAGACAAATCCTGGACAGTGAGGAGATGCAGGAACTGAGAAAGCTGAAGGATGAGTTGGGAGTTATTCTCAAAGAGCTGGCAGAATCTGAGAATGGTCTGGTCCAAAAGAAGCTGTTGGTGAGCAATCTCATCTCAGATGTGGAGCGCCATTTGCAGGGATCAACAATGGAGCTGCTGCAGGATGTGAATGTTATCATAAAAAGGAGTGAAACCTTGGCTCTGAAGAAGCCAAAATCTTTCCCCAAGGAACAGAGGAGAGTGTTTCGAGCTCCTGATCTGAGAGAGATACTGCGTGTGTTTAATGAGCTGACAGATGTGCAACGCTACTGGGTTCAGGTGACCCTGAATTCTCCCAAGAGTGAAAATGTTGTCATTTCTCCGGATCAGAGACAAGTGAGATATGCATCCAGTGACCAATGCTCTAATGCGTATTTGAATGATAATTGTGAAGATTTTGGTGTCCTGGGCTCCCCAGTTATCACATCAGGGAGACATTACTGGGAGGTAGACGTGTCAAAGAAACGTGCCTGGATCTTGGGGGTATGTGATGAAAATATCATAATAGAACCATCAATATTTGGAAATTTTGGAAATGGAAATTGTCAAAATGTTTATTCAAGATATCAACCTAAAAATGGCTATTGGGTCATAGGGTTAAAACATGATTCTGTATATAAAGCCTTTGAGTCTTCCTTCTCAGGTCCTGAGATTTTAACCCTCTCCCTGAGTGTTTCTCCCCATCGTATTGGGATTTTCCTAGACTATGAGGCTCGCACTGTCTTATTTCTCAATGTCACCAACCATGGGTTTCCCATTTATAAATTCTCTTCATGTTCCTTTCCTCAGAACATAGTTCCATATTTCAATCCTATGAACTGTGATGCCCCCATGACTCTGTGCTCACCAAGCTGTTGA

>CowTRIM5-4 Gnomon hmm40150 predicted ORF. This gene prediction from NCBI does not exactly agree with the AC149772 BAC sequence, where there is a frame-shifting mutation in the B30.2-encoding exon 8. This may turn out to be a pseudogene.

ATGGCTTCAGGAATTGTGGTGAACCTACAGCAGGAGGTAACCTGCCCCATTTGCCTGGAGCTTCTGACAGAACCCCTGAGCCTTGACTGTGGCCATAGCTTCTGCCAAGCCTGCATCACTGCAAACAACATGGTGTCCATGAATGACCAAGACGAGGACAGAAGGTGCCCTGTGTGCAGAATTAGTTATGAGCCTGGGAACCTGCGGCCTAATAGGCATGTGGCCAACATAGTACAGAGGCTCAGAGAGGTCAAGTTGAGCCCAGAGGTGGAGCAAGAGAGAAATCTCTGTCTGCGCCATGGAGAGAAACTCATGCTCTTCTGTGAGGAGGACAGGGAAGTCATTTGCTGGCTTTGTGCACGGTCCCAGGAGCACCATGGTCACCACACATTCCTCATGGAAGATGTTGCCCAGGACTATCAGAAGAAACTCCAAGAAGCTCTAGAGAAGCTGAGGGAGAAGCAGCTGGAAGCTGAGGAGTTGGAAGTTAAAGTCAGAGCAGACACTGCTGCCTGGAAGGACCAAATACAACGTGAGAGACAAAATGTCAAGGCAGTGTTTCAGAAACTGAGAAAGAACCTTAAATATGAGGAAGTGAAGGAACTGCAAAAACTGAAGGGTAAGGAGGAAGTTGGTCTACGTAACCTGGCAGACTCTGAGCATGTGCTGATTCAGCAGAGCCAGCTGGTGAGAAATCTCATCTCAGATGTGGAACATCGTTTGAAAGGGTCAACAATGGAGATGCTGCAGGATGTGAACAACATCATGAAAAGGAGTGAAACCTTAACCCTGAAGAAGCCCAGAACTCTCCCCAAGGGACAAAGGAGAATGTGTTCAGCTCTTGATCTGACAGACATACAGCGAGTGTATAATGGGCTGACAGATGTGCAACGCTACTGGGGTAAGGAAACATCACAATGTTATCAAGACCTTGCCATTTCTGAGGACCAGAAGCAATTGAGATATGTGTGCGATTTGGAACCAAACAATATATATAAGAATGATGATTTTGAAGATCATGGTGTCCTGAGCTCCCCAGTTATCACATCACAGAAATATCATTGGGAGGCAGACGTGTCAGAAAAACCTGCCTGGATCCTGGGAGCATATGGTAGAAAACAGCATGGATATAAGATGAAGTTTCTATTTGAAAACAAGGGAAATTGTCAATATGTCTACTGGAGATATCAACCTAAATATAGCTACTGGATTAGAGGGTTAAAAAGAATATGTGAATATGATGCTTTTGATGAGTCTTCTTCCTCAGGTCCCTTGATCTTAACCCTCTCCCTGAGTATTCCTCCTCATTGTATTGGGGTTTTCTTAGACTATAATACTGATGTCTTATTTGTCAATGTCACAAACCAAGGAATTCTCATCTATAAACTCTCTTCATCTTCCTTTACTCAGAAAATATTTCCATATTTTAATCCTGGGAAATGTAATGCTCCCATAACTCTGTGA

>CowTRIM5-5 Gnomon hmm94967 predicted ORF. Also XM_001255273.1| PREDICTED: Bos taurus similar to tripartite motif protein TRIM5 (LOC788122).

ATGATGATTGAGCATGGAGGCGACACGGCTGCTTGGATCGTGGGAACTCTGGACCTGAGAGAGATCCTGAACAATGAGCAATTGAAGGAATGGCAAAACCTGAAGACTGAGGAGGAGGTTGGTCTATGTAATCTGGCTGACTCTGAGAATGAGCTGGTCCAGCAGAGCCAGTTGATGAGAGATCTCATCTCAGATGTGGAGCATCGTTTGCAGGAACCAAAAAACAAGATGCTGCAGACTGAAGAAGGTGTTCTGGAAGCTCCTCAGTTACTGGTGCTGGGAAAACTGTATAACTGCACACATGAGATGAGAACCGAACATTTCCTCCCACCATATACAAAGATCCCTCAACAAATTGAATGA

>CowTRIM5-6 Gnomon hmm39768 gene. Identical to NM_001034798 and DQ381151, mRNA. ATGGCTTCAGGAATCCTGATGAACATACAGGAGGAGGTGACCTGCCCCATTTGCCTGGAGCTTCTGACAGAACCCCTGAGCCTTGACTGTGGCCACAGCTTCTGTCAAGCCTGTATCACTGCAAACAACAAGGAATCCATAATTGGCCAAGAGGGGAAGAGAAGCTGCCCTGTGTGCAGAGTTAGTTTTGAGCCTGGGAACCTGCGGCCTAACAGGCATGTGGCCAATATAGTGCAGAGGCTCCGAGAGGTTAAGGTGAGCCCAGAGGTGGAGCAAGAGAGAAATCTCTGTGCTCACCATGGAGAGAAACTCCAGCTCTTCTGTGAGCAGGATGGGAAGGTCATTTGTTGGCTTTGTGAGCGATCTCAGGAGCACCGAGCTCACAACACATTCCTCATGGAGGAGATTGCGCCAGAGTACCAGAAAAAGCTCCAATCATGTCTGCAGAGGCTGAAGGGGAAAAAACAGGAAGCTGAGGAGTTGGAAATTAAAGTCAGAGAAGAGATGTCTACTTGGAAGATTCAAATAGAAAATGAGATACAAAATGTCCAGGGAAAGTTTACACAACTAAGACAAATCCTGGACAGTGAGGAGATGAAGGAACTGAGAAAGCTGAAGGATGAGTTGGGAGTTATTCTCAAAGAGCTGGCAGAATCTGAGAATGATCTGGTCCAAGAGAAGCTGTTGGTGAGCAATCTCATCTCAGATGTGGAGCGCCATTTGCAGGGATCAACAATGGAGCTGCTGCAGGATGCGAATGTTATCATAAAAAGGAGTGAAACCTTGGCTCTGAAGAAACCAAAATCTTTCCCCAAGGAACAGAGGAGAGTGTTTCGAGTTCCTGATCTGAGACAGACACTGCATGTGTTTAATGAGCTGACAGATGTGCAACGCTACTGGGTTCAGGTGACCCTGAATTCTCCCAAGAGTGAAAATGTTGTCATTTCTCCGGATCAGAGACAAGTGAGATATGCATCCAGTGACCAATGCTATAATGCGTATTGGAATGATAATTATGAAGATTTTGGTGTCCTGGGCTCCCCAGTTATCACATCAGGGAGACATTACTGGGAGGTAGACGTGTCAAAGAAAAGTGCCTGGATCCTGGGGGTATGTGGTGAAAAATGTGAAGATATCATGAAGTTACCATCAAAACTTGGAAATTGTGGAAATGGAAATTGTCAAAATGTTTATTCAAGATATCAACCTAAAAATGGCTATTGGGTCATAGGGTTAAAGCATTATTGTGTATATAATGCCTTTGATGCATCTTCCTTCTCAGATCCTGTGATTTTACCCCTCTCCTTGAGTGTTTCTCTCCATCGTGTTGGGGTTTTCCTAGACTATGAGGCTCACACTGTCTTATTTCTCAATGTCACCAACCATGGGTTTCCCATTTGTCAATTCTCTTCATGTTCCTTTCCTCAGAACATAGTTCCATATTTCAATCCTATGAACTGTGGTGTCCCCATGACTCTGTGCTCACCAAGCTGTTGA

>CowTRIM5-7 Gnomon hmm94585 predicted pseudogene

ATGGCTTCAGGAATTGTGGTGAACCTACAGGGGGAGGTGACCTGCCCCATTTGCCTGCAGCTTCTGACAGAACCCCTGAGCCTTGACTGTGGCCACAGCTTCTGCCAAGCCTGCATCACTGCAAACAACCTGGTGTCCATGAATGACCAAGACGAGGACAGAAGGTGCCCTGTGTGCAGAATTAGTTATGAGCCTGGAAACCTAAGGCCTAATAGGCATGTGGCCAACATAGTACAGAGGCTCAGAGAGGTCAAGTTGAGCCCAGAGGTGGAGCAAGAGAGAAATCTCTGTCTTTGCCATGGAGAGAAACTCATGCTCTTCCGTGAGGAGGACAGGGAAGTCATTTGCTGGCTTTGTGAGCAGTTCCAGGAGCACCATGGTAACCACACATTCCTCATGGAAGATGTTGCCTAGGACTATCAGAAGAAACTCCAAGAAGCTCTAGAGAAGCTGAGGGAGAAGCAGCTGGAAGCTGAGGAATTGGAAGTTAAAGTCAGAGCAGATATTCCTGCCTGGAAGGATCAAATACAACGTGAGAGACAAAATGTCAAGGCAGTGTTTCAAAAACTGAGAAAGAACCTTAAATATGAGGAGGTGAAGGAACTGCAAAAACTGAAGGGTAAGGAGGAANNGTTGGTCTACTACCTGGCAGACTCTGAGCATGTGCTGATCCAGCAGACCCAGCTGGTGAGAAATGCCATCTCAGATGTGGAACATCGTTTGAAAGGATCAACAATGGAAATGCTGCAGGGTGTGAACAACATCATGAAAAGGAGTGAAACCTTAACCCTGAAGAAGCCCAGAACACTCCCCAAGGGATAAAGGAGAATGTGTTCAGCTCTTGATCTGACAGACATACAGTTTATTATTATTATTTCAGGGCTGACAGACGTGCAATGCTACTGGGGACCAGAAGCAATGAGATATGTGAGAGATTTGGAACCAAACAATATATATAAGAATGATGATTTTGAAGATCATGGTGTCCTGGGATCCCCAGTTATCACATCACAGAAATATCACTGGGAGGCAGATGTGTCAGAAAAACCTGCCTGGGTCCTGGGAATATATGGTGGAAAACAGAATGAATATACGATGAAGTTTCTATTTGAAAACAAGGGAAATGACTGTAATACTGATGTCTTATTTGTCAATGTCACAAACCAAGGAATTCTCATCTATAAACTCTCTTCATGTTCCTTTTCTCGGAAAATGTTTCCACATTTCAATCCTGGGAAATGTAATGCTCCTGTAACTGTGATGGCCAAATTCTTGAACTTTCTTAGATTCACACTCACTTCTTTGTAG

>CowTRIM5-8 Gnomon hmm93057 predicted pseudogene

ATGGCTTCAGTAATCCTGGTGAACTTACAGGAGGAAGTGACCTGCCCCATTTTCCTGGAGCTTCTGATAGAATGCTTGAGCCCTGACTGTGGGCAAAGCTTCTACCAAGCCTGCATCACCACTGAAGCACAGCACAAAATAGCCATGTAGGAGAAGGGAGAGAGTAACTCTTTTGTGTGCAGATTCAGTTATGAGCCTTGGAACCTGCGGCCTAATAGGCATGAGGCCAGCATAGTGCAGAAGTTCAGGGAGGTCAAGGGGAGCCTGGAGGAGCAAGACAGAAATCTCTGCACCATGNNGAGAAAACCCCTGCTCTTCTGTGAGGAAGATGGGAAGCTCATTTGCTGGCTTTGCGAGTGGTCCCAGGAGTACCGTGGTCACCACACGTTCCTCATGGAGGACGTTGCCCAACAGTACCAGGAAAGGCTCCAATCATATTTGAAGAGGTTGAAGAGGGAGAAGCAGGAAGCTGCGAAGATGGGATCTTATTTCAAAAAAGAGATATCTACCTGGAAGGCAAGAATCAAGTATAACATGAGACAAAATGTCCAGGCAGAGTTTAAAAAANNCAGAGTGATCCTGGACAGTGAGGAGCTGAAGTGACTGCAAAAGTTGAGAAATGAAGAGAAAGTTGGTCTACAGAACATGGCAAATTCTGAGAATGGGATGGTCCAGTAGAGTCAATTGGTGNNACATCTCATCTCAGATCTGGAGCATCATTTGCAGGGATCAGCAATGGAGATGCTGCAGATAAGACTTGGAAAGAAGTCTGAGGATCCAAGCTATGCTTTGTTAATTTATTGA

>CowTRIM5-9 Gnomon hmm10462 predicted ORF. Contains no introns. Also XM_001250593 PREDICTED: Bos taurus similar to tripartite motif protein TRIM5 (LOC782527)

AGCCTGGGAACCTGCGGCCTAACAGGCATGTGGCCAATATAGTGCAGAGaCTCtGAGAGGTTAAGGTGAGCCCAGAGGTGGAGCAAGAGAGAAATCTCTGTGCTCACCATGGAGAGAAACTCCAcCTCTTCTGTGAGCAGGATGGGAAGGTCATTTGTTGGCTTTGcGAGtGATCTCAGGAGCACCGAGgTCACAACACATTCCTCATGGAaGAGATTGtGCCAGAGTACCAGAAAAAGCTCCAATCATGTCTGCAGAGGCTGAAGGGGAAAAAACAGGAAGCTGAGGAGTTGGAAATTAAAGTCAGAGAAGAGATGTCTACTTGGAAGATTCAAATAcAAAATGAGATACAAAATGTCCAGGGAAAGTTTACACAACTAAGACAAATCCTGGACAaTGAGGAGATtAAGGAACTGAGAAAaCTGAAGGATGAGTTGGGAGTTATTCTCAAAGAGCTGGCAGgATCTaAGAATGATCTGGTCCAAGAGAAGCTaTTGGTGAGCAATCTCATCTCAGATGTGGAGCGCCATTTGCAGGGATCAACAgTGGAGCTGCTGCAGGATGtGAATGTTATCATgAAAAGGAGTGAAACCTTGGCTCTGAAGAAgCCAAAATCTTTCCCCAAGGAACAGAGGAGAGTGTTTCGAGcTCCTGATCTGAGAgAGAtACTGCATGTGTTTAATGAGCTGACAGATGTGCAACGCTACTGGGTTCAGGTGACCCTGAATTCTCCCAAGtGTGcAAATGTTGTCATTTCTCCGGATCAGAcACAAGTtAGATATGCATCCAGTGACCAAgGCTATAATGCaTAcTtGAATtATAATTATGAAacTTTTGGTGTCCTGGGCTCCCCAGTTATCACATCAGGGAGACATTACTGGGAGGTAGACGTGTCAAAGAAAAGTGCCTGGATCCTGGGGGTATGTGGTGAAAAATGTGAAaATAcCATGAtagTACCATCAgtAtTTGGAAATTGaGGAAATGgaaattatccaattggtttttcaggatataaacctaaaaatagaaattgtgGAAATTGTCAcAATGTTTATTCcAGATATCAACCTAAAAATGGCTATTGGGTCATAGGGTTAAAaCATgATTcTGTATATAATGCCTTTGAgTCTTCCTTCTCAGgTCCTGTGATTTTAagCCTCTCCcTGAGTGTTTCTCcCCATCGTGTTGGGGTTTTCCTAGACTATGAGGCTCACACTGTCTTATTTCTCAATGTCACCAACCATGGGTTTCCCATTTaTacATTCTCTTCATGTTCCTTTCCTCAGAACATAGTTCCATATTTCAATCCTATGAACTGTGGTGcCCCCATGACTCTGTtCTCACCAAGCcGTTGA

>CowTRIM34 NM_001046461, mRNA. Same as BC114124

ATGGCTTTAAATACTGTGATGAACCTACAGGAAGAGGTATCCTGTCCCATCTGCAGGGAGCTTTTAACAGAACCCCTAAGTCTAGGCTGTGGTCACAGCTTCTGCCAGACTTGCATCACTAACAAGGAGACAGACATCAGCCTAGGAGGGGACAGCAGCTGTCCTGTGTGTGGTACGCGGTACTCACTTGGAAACCTGTGGCCTAATCTGCACCTGGCCAACATAGTAGAGAGACTCAGAAAGGTCAAGCTGAGCCCAGAGGAGGGGCAGAAGACAGGTCTCTGTGTGCACCATGAAGAGAAACTCCTACTCTTCTGTAAGGAAGACAGAAAAGTCATTTGTCGTCTTTGTGAACGATCTCAGGAGCACCATGGTCACCACACATTCCTCATGGAAGAGGCACTCAAGGAAAGTCAGGAGATGCTCCAAGCAGCTCTGACGAGGCTGAGGAAGGAGCAGCAGAAGGCTGAGAAGTTGGAAGCTGACATCAGAGAAGAGAGAACTTCCTGGAAGTATCAGATTCAGACTGAGAGACAAAGGATACAAACAGAGTTTAATCAGCTTAGAAGCATCCTAGACAGTGAGGAGCGAAGAGAATTGCAGAAATTGGAGGAAGAGGAGAAGAAGACCCTGGATAGCTTGGCTGTGGCTGAGGCTGAGCTGGTTCAGCAGGGCGAGCTGCTGAAGGAGCTCATCTCAGATCTGGAGCGCCGCAGTGAATGGTCAACTGTGGAGCTGCTGCAGGACATGAGTGGAATCATGAAATGGAGTGAGATCTGGACACTGAAGAAGCCAAAAACGGTTTCCAAGAAACTGAAGAATGTATTCCGTGTTCCAGATCTGCGTGGAATGCTGCACACGTTTAAAGACCTAACACGCGTCCAGTGCCACTGGGTGGACATCACATTGAATCCATTCAACCTAAATTTGAATCTTGTCCTTTCAGAAGATCAGAGACAAGTGCTATCTGTGCCAATATGGCCTGTTAAGTATTATAATTATGGTATCTTGGGCTGCCAGTATTTCTCCTCAGGGAAACACTACTGGGAAATAGATGTATCCGAGAAGAATGCCTGGATCCTAGGGGTATACTGTCGAATACGCTCCTGTAATATAAAGTTTGCTGTTCAACAAAGCACGAGTTATGAAAATGCTTACTCCATATACAGACCTCAATTTGGTTACTGGGTTATAGGGTTAAAGGATAAATTTAGATATGAAGCCTTTGAGGACTCTTCCACCACTCATCCTGACTCAAGAGTCTTAACTCTTTCCATGACTGTTCCTCCCCGTCATGTTGGTGTTTTTCTAGACTATGAAGCAGGCACTGTCTCATTTTTCAATGTCACAAACCATGGGTCACTCATCTATAAGTTCTCTAAGTGCAACTTTTCTCGGAATGCCTATCCATATTTCAATCCTTGGGACTGTCCGGCTCCCATGACACTGTGCCCTCCAAGTTCGTGA

>CowTRIM6 BC108121 Note Genbank mis-annotation as TRIM22.

ATGACTTCAGCAGTCCTGGTGGACATCCAAGATGAAGTGACCTGCCCCATCTGCCTGGAGCTCCTGACCGAACCCCTGAGCATAGACTGTGGACACAGCTTCTGCCAAGCCTGCATCACTGCAGACAACAAGGAGTCCATGCCTGGCCAGGAGGGGCAGAGCAGGTGTCCTGTGTGCCAGACCAGCTACTGGCTTGGGAACCTGCGGCCCAATCGGCACCTGGCCAACATAGCCGAGAGGCTCAGGGAGGTGGTGGTGGGCTCAGGGAAGCAGCTGAAGGTGATTCTTTGTGCGCACCATGGAGAGAAACTCCAGCTTTTCTGTGAGGAGGATGGGAAGCTCATTTGCTGGCTTTGCGAGCGGTCTCAGGAGCACCGTGGTCACCACACGTTCCTCATGGAGGAGATAGCCCAGGAGTACCAGGAGAAGTTCCAGGAGTCTCTGAAGAAGCTGAGGCAAGAGCAGCAGGAAGCCGAGAAATTAGCAGCTGTTATCAGAGAGAAGAGGATATCCTGGAAGAATCAGATGGAACCTGAGAGACACAGGATCCAGAAACAGTTTGATCACTTGAGGAGCATCCTGGACAAAGAGGAACAGCGACAGCTGAAGAAGCTGGAGGAGGAGGAAAGGAGAGGGCTGAACATCATAGCAGAAGCTGAGGTCGAGCTGGTCCAGCAGAGCCAGTCCCTGAGAGAGCTCATCTCAGACCTGGAGCACCGTTGTCAGGGGTCAGCAACAGAGCTCCTGCAGGATGTGAGTGACGTTGCGAAAAGGAGTGAGTTCTGGACCCTGAAGAAGCCAGAGGCTCTCCCCACGAAGCTGAAGAGTATGTTTCGAGCTCCAGATCTGAAAAAGATGCTTCGAGTGTTTAGAGGTGAGGAGTTTCAGGAAACAGAGTTTGGATGGGGGATCATGGTAGTAGTAGGAAATGAAAAGAATGGAGGGCATGGTTGGTATTTGGGAACAGTGGCAGAGGGAGCAACACAATATGCTGAGGAAGATGCGGTTCATACTTAA
